# OptiPrep™ Density Gradient Solutions for Mammalian Organelles

**DOI:** 10.1100/tsw.2002.840

**Published:** 2002-05-29

**Authors:** John Graham

**Affiliations:** School of Biomolecular Sciences, Liverpool John Moores University, UK

**Keywords:** gradient solutions, OptiPrep™, iodixanol, density, osmolality, pH, mammalian nuclei, mammalian organelles

## Abstract

Any density gradient for the isolation of mammalian organelles should ideally only expose the sedimenting biological particles to an increasing concentration of the gradient solute. Thus they will experience only an increasing density and viscosity, other parameters such as osmolality, pH, ionic strength and the concentration of important additives (such as EDTA and DTT) should remain as close to constant as possible. This Protocol Article describes the strategies for the dilution of OptiPrep™ in order to prepare such solutions for mammalian organelles and membranes.

**METHOD TYPE:** extraction, isolation, purification and separation

**SUB METHOD TYPE:** centrifugation

## OSMOLALITY

The observed osmolality of OptiPrep™ depends on the mode of measurement (freezing point or vapour pressure). The former provides a value of approx 260 mOsm, the latter approx. 170 mOsm. Moreover, irregular freezing of the sample sometimes makes measurement of the freezing point difficult. However, when OptiPrep™ is diluted with water to 50% iodixanol, values of approx. 195 mOsm are consistently obtained. More importantly, OptiPrep™ can be diluted with a buffered iso-osmotic solution to provide a working solution that is also iso-osmotic.

## pH

Because it is a solution in water with no additives, its pH (4-6) is that of water. The actual pH is unimportant, because when OptiPrep™ is mixed with a standard ionic or organic buffer, the pH of the buffer is unaffected by the iodixanol: any changes in pH are the same as those that would occur during dilution of the buffer with water.

## HANDLING OptiPrep™

Prolonged exposure (several months) of iodixanol solutions to sunlight will cause a slow release of iodine; OptiPrep™ should therefore be stored away from strong sunlight. On standing, iodixanol may "settle out" of concentrated solutions; always shake the bottle of OptiPrep™ before use.

## PREPARATION OF DENSITY SOLUTIONS

The recommended procedure for the production of density gradient solutions or for adjustment of the density of organelle suspensions is to use a Working Solution whose composition is compatible with the particles to be separated. The following methodology is based on the use of 0.25 M sucrose, 1 mM EDTA, 10 mM Tris-HCl, pH 7.4 as homogenization medium (HM).

**Prepare a 50% (w/v) iodixanol Working Solution** as follows: mix 5 vol of OptiPrep™ with 1 vol of 0.25 M sucrose, 6mM EDTA, 60 mM Tris-HCl, pH 7.4.

**Density solutions** are then produced from the **Working Solution** (WS) according to Table 1.

**The osmolality** of all the dilutions is in the range 295–310 mOsm. The use of alternative organic buffers at similar concentrations will have no significant effect on the density and osmolality of the Working Solution.

The concentration of buffer in all of the gradient solutions will be the same as in the HM. If the concentration of any other additive (e.g., DTT or a nonionic detergent) needs to be kept constant in the gradient, this can also be added to the OptiPrep™ diluent at the appropriate concentration.

## ADJUSTING THE DENSITY OF A SAMPLE

The 50% (w/v) iodixanol Working Solution is also suitable for adding to a homogenate or differential centrifugation fraction (suspended in 0.25 M sucrose) in order to adjust its density. Although in many cases it is acceptable to add OptiPrep™ directly.

## OTHER MODES OF SOLUTION PREPARATION

It may be permissible to produce density solutions simply by diluting OptiPrep™ with homogenization medium. The osmolality will be satisfactory but the concentration of buffer and additives will decrease as the iodixanol concentration increases.

## OTHER OSMOTIC BALANCERS

Occasionally mannitol (or sorbitol) may be preferred over sucrose as an osmotic balancer for mammalian systems. Mannitol in particular is widely used in media for the isolation of mitochondria and sometimes it is used for suspending cells when a nonionic medium is required. These osmotic balancers are also preferred for many nonmammalian systems[1].

**TABLE 1**
**Density (ρ) And Refractive Index (η) Of Iodixanol Solutions: Dilution Of 50% Iodixanol Working Solution (Ws) With Hm**

| % w/v iodixanol | η | ρ (g/ml) | WS (ml) | HM (ml) |
|---|---|---|---|---|
| 10.00 | 1.3589 | 1.078 | 2.00 | 8.00 |
| 12.00 | 1.3617 | 1.088 | 2.40 | 7.60 |
| 14.00 | 1.3645 | 1.098 | 2.80 | 7.20 |
| 16.00 | 1.3673 | 1.107 | 3.20 | 6.80 |
| 18.00 | 1.3701 | 1.117 | 3.60 | 6.40 |
| 20.00 | 1.3729 | 1.127 | 4.00 | 6.00 |
| 22.00 | 1.3757 | 1.136 | 4.40 | 5.60 |
| 24.00 | 1.3785 | 1.146 | 4.80 | 5.20 |
| 26.00 | 1.3813 | 1.156 | 5.20 | 4.80 |
| 28.00 | 1.3840 | 1.165 | 5.60 | 4.40 |
| 30.00 | 1.3868 | 1.175 | 6.00 | 4.00 |
| 32.00 | 1.3896 | 1.185 | 6.40 | 3.60 |
| 34.00 | 1.3924 | 1.194 | 6.80 | 3.20 |
| 36.00 | 1.3952 | 1.204 | 7.20 | 2.80 |
| 38.00 | 1.3980 | 1.214 | 7.60 | 2.40 |
| 40.00 | 1.4008 | 1.223 | 8.00 | 2.00 |
| 42.00 | 1.4036 | 1.233 | 8.40 | 1.60 |
| 44.00 | 1.4064 | 1.243 | 8.80 | 1.20 |
| 46.00 | 1.4091 | 1.252 | 9.20 | 0.80 |
| 48.00 | 1.4119 | 1.262 | 9.60 | 0.40 |
| 50.00 | 1.4147 | 1.272 | — | — |

*Note*: Refractive index and density are related by the following simple equation: $\rho = 3.4713\eta - 3.6393$.

Iso-osmotic density solutions based on an HM containing 4.4% (w/v) mannitol (or sorbitol), 10 mM Tris-HCl, pH 7.4 ($\rho = 1.015$ g/ml) are produced in the same manner as those based on 0.25 M sucrose. A 50% (w/v) iodixanol Working Solution (WS) is produced by diluting 5 vol of OptiPrep™ with 1 vol of 4.4% (w/v) mannitol, 60 mM Tris-HCl, pH 7.4. This is then diluted further with HM. The properties of a few selected dilutions are given in Table 2.

**The osmolality** of the solutions is in the range 290–310 mOsm.

**TABLE 2**
**Density (ρ) and Refractive Index (η) of Mannitol-Containing Iodixanol Solutions: Dilution of 50% Iodixanol Working Solution (WS) with HM**

| % w/v iodixanol | η | ρ (g/ml) | WS (ml) | HM (ml) |
|---|---|---|---|---|
| 10 | 1.3548 | 1.065 | 2.00 | 8.00 |
| 20 | 1.3796 | 1.116 | 4.00 | 6..00 |
| 30 | 1.3844 | 1.167 | 6.00 | 4.00 |
| 40 | 1.3991 | 1.218 | 8.00 | 2.00 |
| 50 | 1.4139 | 1.269 | | |

## PREPARATION OF DENSITY SOLUTIONS FOR NUCLEI

The majority of homogenization solutions for the isolation of nuclei contain KCl and $MgCl_2$ as opposed to EDTA. An homogenization medium (HM) of 0.25 M sucrose, 25 mM KCl, 5 mM $MgCl_2$, 20 mM Tris-HCl, pH 7.8 is often recommended.

Mix 5 vols of OptiPrep™ with 1 vol of 150 mM KCl, 30 mM $MgCl_2$, 120 mM Tris-HCl, pH 7.8, to produce a Working Solution (WS) with a density of 1.269 g/ml and osmolality of 320 mOsm.

Dilute the WS with HM (ρ = 1.033 g/ml) to provide solutions of the appropriate density.

The density of any gradient solution can be calculated using Eq. 1.

$$D = \frac{Vd + V_1 d_1}{V + V_1} \quad (1)$$

*D* = Density of mixture; *V* = volume of OptiPrep™; *d* = density of OptiPrep™; $V_1$ = volume of diluent; $d_1$ = density of diluent.

## ACKNOWLEDGEMENTS

The author and TheScientificWorld wish to thank Axis-Shield PoC, AS, Oslo, Norway for their kind permission to adapt OptiPrep™ Application Sheet S1 in the preparation of this Protocol Article.
